# Sleep and behavioral problems in Down syndrome: differences between school age and adolescence

**DOI:** 10.3389/fpsyt.2023.1193176

**Published:** 2023-06-09

**Authors:** Elisa Fucà, Floriana Costanzo, Laura Celestini, Paolo Galassi, Alberto Villani, Diletta Valentini, Stefano Vicari

**Affiliations:** ^1^Child and Adolescent Neuropsychiatry Unit, Bambino Gesù Children's Hospital, IRCCS, Rome, Italy; ^2^Pediatric Unit, Pediatric Emergency Department, Bambino Gesù Children's Hospital, IRCCS, Rome, Italy; ^3^Department of Life Science and Public Health, Catholic University of the Sacred Heart, Rome, Italy

**Keywords:** trisomy 21, Child Behavior Checklist, sleep disorders, Sleep Disturbance Scale for Children, psychopathology

## Abstract

**Background:**

Individuals with Down syndrome (DS) are at risk of developing sleep problems. In spite of the well-established knowledge on the presence of sleep difficulties in DS individuals and the associated emotional and behavioral problems, less is known about the possible differences in the kind of associations between sleep and emotional/behavioral problems across different age ranges.

**Methods:**

In this retrospective study, we included 289 participants with DS aged 6–18 years with the aims to explore differences in the distribution of sleep problems between specific age groups (school age vs. adolescence) and to identify specific age-based associations between sleep problems and emotional/behavioral problems.

**Results:**

Some differences in the distribution of sleep problems have emerged between age groups. Moreover, differences in the patterns of association between emotional/behavioral difficulties and sleep problems-in particular, sleep-related breathing difficulties and parasomnias-have been observed. However, sleep-wake transition disorders and excessive daily somnolence appear to be related to emotional and behavioral problems (both internalizing and externalizing), in general, across school age and adolescence.

**Discussion:**

These results remark the importance of appropriate neuropsychiatric and psychological evaluation taking into account the age-specific needs and features of individuals with DS.

## Introduction

Sleep is crucial for healthy development. However, children frequently experience sleep problems, such as insomnia, nighttime waking, nightmares, and inconsistent and maladaptive bedtime routines (1–3). The estimated prevalence of sleep disorders in pediatric age may vary extensively, depending upon differences in the definitions and methods of assessment (4). It ranges from 37.6 to 62% for children (5, 6) and from 20 to 26% for adolescents (7, 8). In particular, sleep deprivation in adolescents is common: the reported percentages of adolescents getting < 8 h of sleep on weeknights range from 62 to 72.7% (9, 10). The clinical manifestations of sleep disorders vary greatly among different age groups. Indeed, during the first years of life, the most frequent sleep difficulties are linked with problems falling asleep, frequent nocturnal awakenings, parasomnias, and sleep-disordered breathing. In preschoolers, disorders related to inadequate sleep hygiene can emerge. Finally, in adolescence, frequently occurring sleep disorders are related to circadian issues or excessive movement during sleep, e.g., restless leg syndrome (11).

Sleep problems in childhood and adolescence are associated with poorer scholastic achievement (12, 13), poorer student–teacher relationship quality (14), and worse socioemotional functioning (15, 16) both acutely and throughout development (17, 18). Sleep difficulties in childhood and adolescence have also been linked to a variety of externalizing problems (19, 20) as well as internalizing problems (20–22). Finally, sleep difficulties in a child might have a negative effect on the wellbeing of caregivers (23).

In children with neurodevelopmental disorders, such as intellectual disability or autism spectrum disorder, the prevalence of sleep disorders is higher than the typically developing population (TD), with an estimated prevalence reported to be as high as 80% (24, 25).

Among pediatric populations with neurodevelopmental disorders, sleep is an important concern, especially for individuals with Down syndrome (DS). Indeed, given the alterations in craniofacial and oral musculature associated with the syndrome, individuals with DS are particularly prone to obstructive sleep apnea (OSA), affecting 69–76% of children with DS (26). In addition, children with DS exhibit a different sleep architecture in comparison with children without DS: these differences have been shown to continue throughout adolescence and early adulthood (27). Individuals with DS exhibit REM sleep abnormalities as well as greater sleep fragmentation, exhibit more time awake after sleep onset, and exhibit lower sleep efficiency than TD (28–30). Similar to TD, sleep difficulties and sleep disorders in children and adolescents with DS have been associated with a number of cognitive, emotional, and behavioral problems, including both internalizing and externalizing symptoms, such as attention problems, withdrawal, and hyperkinesia (31–34).

In spite of the well-established knowledge on the presence of sleep difficulties in DS and the associated emotional and behavioral problems, less is known about the possible differences in the kind of associations between sleep and emotional/behavioral problems across different age ranges. This is crucial, as adolescents with DS exhibit a significant shift in the psychopathological symptoms, with a significant decrease in externalizing and an increase in internalizing problems, such as depression and withdrawal (35–37). Given that sleep problems take different configurations among different age groups, it is of interest to investigate the presence of specific patterns of associations between sleep and behavioral problems in DS among different age groups.

Therefore, the present study had two specific research questions:

- Are there any differences in the distribution of sleep problems between school-age children and adolescents with DS?
- Is it possible to identify specific age-based associations between sleep problems and emotional/behavior problems in children vs. adolescents with DS?

A deep understanding of the nature and development of sleep problems and their relationship with emotional and behavioral difficulties in youth with DS may provide pivotal information for early screening, prevention, and treatment of sleep problems in such a crucial life period.

## Materials and methods

### Participants

A total of 289 children and adolescents with DS (174 boys, 115 girls) ranging in age between 6 and 18.11 years of age (mean 11.58 ± 3.5 years) were included in the study. The sample was divided into two groups: school-age children (6–11 years of age; *N* = 161; mean age: 8.93 ± 1.73 years; 89 boys, 72 girls) and adolescents (12–18 years of age; *N* = 128; mean age: 14.91 ± 1.2 years; 85 boys, 43 girls). Data were collected from a file review of children and adolescents with DS who were referred for a clinical evaluation at the Pediatric Unit and/or the Child and Adolescent Neuropsychiatry Unit of a pediatric hospital between January and October 2022. The selection criteria included the diagnosis of DS based on the analysis of the karyotype and age ranging between 6 and 18 years. The exclusion criteria were age < 6 or >18 years; the ascertained presence or the clinical suspect of neurological conditions, such as West syndrome and epilepsy; and the language barrier hampering the compilation of a questionnaire for parents.

### Procedure

This was a cross-sectional study. Data were retrospectively collected from a file review of children and adolescents with DS referred for a clinical evaluation at the Pediatric Unit and/or the Child and Adolescent Neuropsychiatry Unit of a pediatric hospital between January and October 2022. Young people with DS underwent a pediatric and/or a neuropsychological evaluation. As part of the clinic visit, caregivers typically completed parent-reported measures to investigate the presence of sleep difficulties and psychopathological questionnaires regarding their child. Due to the retrospective design, data were collected from the hospital records and clinic charts and the de-identified data were analyzed. All parents signed a written informed consent for data use for research purposes and a privacy statement that ensures that their data will be kept confidential. The study was conducted according to the guidelines of the Declaration of Helsinki.

### Measures

Sleep disturbances were assessed by means of the Sleep Disturbance Scale for Children—SDSC (38), a questionnaire that has demonstrated through validation an adequate level of internal consistency, good test–retest reliability, and availability of normative data. The SDSC explores the presence of sleep disorders during the last 6 months and contains 26 items with Likert scale values ranging from 1 to 5. The questionnaire consists of 26 items subdivided into six sleep disorder subscales: disorders of initiating and maintaining sleep (DIMS; e.g., sleep duration and sleep latency), sleep breathing disorders (SBD; e.g., snoring and breathing problems), disorders of arousal (DA; e.g., sleepwalking and nightmares), sleep–wake transition disorders (SWTD; e.g., bruxism and sleeptalking), disorders of excessive somnolence (DOES; e.g., sleep attacks and daytime somnolence), and sleep hyperhidrosis (SHY; e.g., night sweating). The sum of scores provides a total sleep score with a possible range from 26 to 130; a T-score of more than 70 is considered pathological.

Emotional and behavioral problems were evaluated by means of the Child Behavior Checklist—CBCL (39). The CBCL is a parent/caregiver report form to screen for emotional, behavioral, and social problems. The school-age version (CBCL/6–18) is for children aged between 6 and 18 years. It is composed of eight Empirically Based Syndrome Scales (Anxious/Depressed, Withdrawn/Depressed, Somatic Complaints, Social Problems, Thought Problems, Attention Problems, Rule-Breaking Behavior, and Aggressive Behavior), three general domains (Total, Internalizing, and Externalizing problems), and six Diagnostic and Statistical Manual of Mental Disorders (DSM)-oriented scales (Affective Problems, Anxiety Problems, Somatic Problems, Attention Deficit/Hyperactivity Problems, Oppositional Defiant Problems, and Conduct Problems). For the current study, Empirically Based Syndrome Scales were considered.

### Data analysis

Descriptive statistics were used to analyze the demographic characteristics of the whole sample. Correlation analyses were used to investigate the possible association between sleep problems and behavioral and emotional problems. The chi-squared test was used to determine the non-parametric variables and *t*-tests, and repeated-measures analysis of variance (ANOVA) was computed on SDSC scores to detect differences between sex/age groups and on CBCL scores to investigate differences according to the presence/absence of sleep problems per each age group. Statistical tests were used with a significance level of *p* of < 0.05.

## Results

### Distribution of sleep problems and emotional and behavioral problems: differences between school-age children and adolescents

To explore differences in the distribution of sleep problems between school-age children and adolescents, we explored the percentage of individuals exhibiting clinical/borderline scores at SDSC within each group. The results are reported in Table 1. The chi-square statistics revealed significant group differences in the distribution of clinical/borderline scores and scores in the DA (*p* = 0.006) and SWTD (*p* = 0.047) subscales.

**Table 1:** Distribution of scores in the clinical/borderline range at the SDSC subscales (%).

	**School-age children (*N* = 161)**	**Adolescents (*N* = 128)**
DIMS	32	32
SBD	40	33
DA	30	14
SWTD	38.5	25
DOES	14	20
SHY	12	8

DIMS, disorders in initiating and maintaining sleep; SBD, sleep breathing disorders; DA, disorders of arousal; SWTD, sleep–wake transition disorders; DOES, disorders of excessive somnolence; SHY, sleep hyperhidrosis.

Sex differences in the SDSC scores were explored within each age group. With regard to the group of school-age participants, there were no statistically significant differences in any of the SDSC scales (all *p* > 0.05). With regard to the group of adolescents, significant differences emerged on the SBD scale, with boys exhibiting significantly higher scores than girls (62.38 ± 16.98 and 54.81 ± 11.34, respectively; *p* = 0.009).

**Figure 1:**
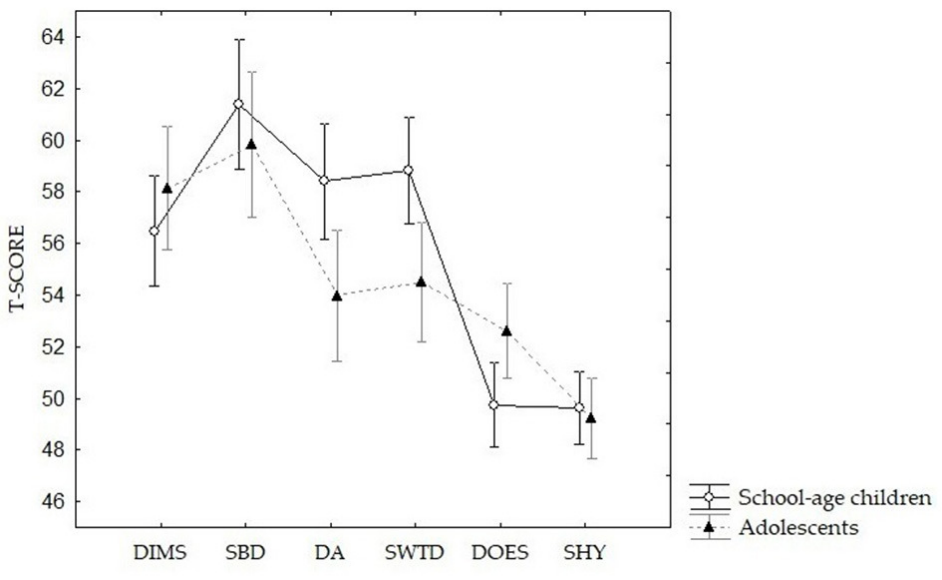
Differences between school-age children (*N* = 161) and adolescents (*N* = 128) on the SDSC scores (T-scores). DIMS, disorders in initiating and maintaining sleep; SBD, sleep breathing disorders; DA, disorders of arousal; SWTD, sleep–wake transition disorders; DOES, disorders of excessive somnolence; SHY, sleep hyperhidrosis.

A repeated-measures ANOVA on SDSC, with age group (school-age children/adolescents) as the between-subject factor and SDSC scores as within-subject factors, was performed, showing a significant effect of the SDSC subscales, highlighting higher scores for SBD compared with the other subscales (all *p* ≤ 0.002) and lower scores for DOES and SHY compared with the other subscales (all *p* ≤ 0.001), irrespective of the group. Moreover, an interaction between age group and SDSC scores emerged, F_(5, 143)_ = 5.2848, *p* < 0.001, ηp^2^ = 0.02 (see Figure 1), with lower scores for DA and SWTD subscales in the adolescent group compared with the school-age group. However, such differences did not reach statistical significance on *post-hoc* analysis (Tukey's HSD test; all *p* > 0.05).

Differences in emotional and behavioral problems between age groups were also explored. Repeated-measures ANOVA with age group (school-age children/adolescents) as the between-subject factor and CBCL subscales as the within-subject factors was performed, showing a significant interaction F_(7, 2009)_ = 9.6514, *p* < 0.001, ηp^2^ = 0.03. *Post-hoc* analysis (Tukey's HSD test) revealed significant differences on the Withdrawn/Depressed scale (57.84 ± 8.74 and 61.47 ± 9.06 for school-age children and adolescents, respectively; *p* = 0.002).

### Association between sleep difficulties and emotional/behavioral problems

With the aim to explore the presence of associations between sleep difficulties and emotional/behavioral problems in each group, we performed correlation analyses (Pearson correlation). The results are summarized in Table 2.

**Table 2:** Association between sleep difficulties and emotional/behavioral problems.

**School-age children (*****N*** = **161)**								
	**Anxious/depressed**	**Withdrawn/depressed**	**Somatic complaints**	**Social problems**	**Thought problems**	**Attention problems**	**Rule-breaking behavior**	**Aggressive behavior**
DIMS	**0.233** ^*^	0.071	**0.337** ^*^	0.213	**0.3728** ^*^	**0.251** ^*^	**0.386** ^*^	**0.224** ^*^
SBD	0.114	0.112	**0.292** ^*^	0.132	0.216	**0.251** ^*^	0.213	0.097
DA	0.138	0.008	**0.229** ^*^	−0.05	0.162	0.097	0.144	−0.083
SWTD	0.185	0.045	**0.362** ^*^	**0.277** ^*^	**0.357** ^*^	**0.329** ^*^	**0.393** ^*^	0.19
DOES	**0.36** ^*^	0.093	**0.332** ^*^	**0.251** ^*^	**0.444** ^*^	**0.324** ^*^	**0.387** ^*^	**0.226** ^*^
SHY	0.009	0.04	0.037	−0.004	0.154	0.176	0.136	0.05
	**Adolescents (*****N*** = **128)**							
	**Anxious/depressed**	**Withdrawn/depressed**	**Somatic complaints**	**Social problems**	**Thought problems**	**Attention problems**	**Rule-breaking behavior**	**Aggressive behavior**
DIMS	**0.263** ^*^	**0.252** ^*^	0.238	**0.313** ^*^	**0.447** ^*^	**0.38** ^*^	**0.292** ^*^	**0.415** ^*^
SBD	0.093	0.209	0.215	0.198	0.157	0.092	0.166	0.139
DA	0.083	0.177	0.199	0.141	**0.266** ^*^	**0.029** ^*^	0.183	**0.34** ^*^
SWTD	**0.245** ^*^	0.236	**0.255** ^*^	**0.411** ^*^	**0.421** ^*^	**0.487** ^*^	**0.42** ^*^	**0.504** ^*^
DOES	**0.272** ^*^	**0.311** ^*^	**0.339** ^*^	**0.299** ^*^	**0.355** ^*^	**0.321** ^*^	**0.32** ^*^	**0.246** ^*^
SHY	0.156	0.244	0.172	0.128	0.117	0.168	0.18	0.184

^*^Survived after Bonferroni correction with a *p*-value of < 0.006.

With the aim to better characterize age group-related differences in the association between sleep problems and emotional/behavioral problems, repeated-measures ANOVA on CBCL, with Sleep Group (Sleep problems/No sleep problems) as the between-subject factor and CBCL subscales as the within-subject factors, was performed for each age group. Within each age group, we distinguished among children who displayed SDSC Total score in the clinical/borderline range and children who exhibited SDSC Total Score in the normal range. With respect to school-age children, a significant Group Effect (*p* < 0.001) emerged, underlining that participants with clinical/subclinical scores at SDSC exhibited higher scores at CBCL subscales. Moreover, a Subscale effect was detected (*p* < 0.001), with higher scores on Social and Attention problems subscales in comparison with the other CBCL subscales (all *p* < 0.001). Finally, an interaction effect emerged F_(7, 111)_ = 2.152, *p* = 0.036, ηp^2^ = 0.05. *Post-hoc* analysis (Tukey's HSD test) revealed significant differences in several CBCL scales between school-age children with sleep problems and school-age children who exhibited SDSC Total Score in the normal range. In particular, significant differences emerged in the Somatic Complaints scale (54.89 ± 5.29 and 60.6 ± 7.84 for children without and with sleep problems, respectively; *p* < 0.001), Thought Problems (55.83 ± 6.37 and 62.51 ± 8.74, respectively; *p* < 0.001), Attention Problems (60.26 ± 7.16 and 64.75 ± 7.72, respectively; *p* = 0.01), and Rule-Breaking Behavior (55.45 ± 5 and 59.54 ± 6.46, respectively; *p* = 0.036). With respect to adolescents, a significant Group Effect (*p* < 0.001) emerged, underlining that participants with clinical/subclinical scores at SDSC exhibited higher scores at CBCL subscales. Moreover, a Subscale effect was detected (*p* < 0.001), with high scores on Social Problems and Withdrawn/Depressed subscales. However, a significant interaction did not emerge F_(7, 882)_ = 1.721, *p* = 0.1, ηp^2^ = 0.01.

## Discussion

The first aim of the current study was to investigate the presence of differences in the distribution of sleep problems between school-age children and adolescents with DS.

The results showed a significant group effect. School-age children with DS exhibited a higher percentage of clinical/borderline scores at the DA scale than adolescents did (30 vs. 14%), as well as at the SWTD subscales (38.5 vs. 25%). The DA and SWTD subscales of the SDSC reflect parasomnias (38). These prevalence rates are higher than those reported in TD: for example, the total prevalence of night terrors was estimated to be 17.3% in the age group of 3–13 years, while the peak prevalence of sleepwalking, which occurs around age 10 years, was estimated to be 13% (40). However, our findings are in line with evidence on the general population, indicating that parasomnias tend to decrease across the development of young people and in adulthood (40). Although scant evidence is available on the presence of parasomnias and their most frequent types isolated in youth with DS (41), a similar trend has also been observed for such a population. For example, Maris and colleagues (42) reported a negative association between parasomnias and age in a sample of 54 children with DS. Similarly, a decrease in sleep bruxism starting from 12 years of age has been reported in a sample of 57 young people with DS (43). A subsequent systematic review corroborated this trend (44). However, another study failed to detect statistically significant differences among children aged between 4 and 12 years and children aged ≥ 13 years in the prevalence of parasomnias in a sample of 58 individuals with DS (45). Our results confirm the previous findings about the presence of an age-related decrease in parasomnias by examining the prevalence of arousal disorder in a large sample of youth with DS as measured through the SDSC. These results suggest that, although parasomnias occur more frequently than TD, they are usually self-resolving in children with DS. However, even if benign, parasomnias may have negative consequences on a child's quality of life (40). Therefore, when a child with DS exhibits parasomnias, it is crucial to provide a proper evaluation of the impact of such disturbances to provide adequate interventions by reducing negative consequences on quality of life.

However, adolescents exhibited a slight decrease in SBD-related problems. SBD-related problems, especially OSA, represent a major concern in children with DS (26), associated with a range of poorer outcomes in language, working memory, emotional control, and executive function (26, 46, 47). Our results are consistent with previous literature, reporting a higher prevalence of SBD-related problems in both children (42) and adolescents (48, 49) with DS than TD. The higher prevalence of OSA and SBD-related problems in DS is probably linked with the presence of several risk factors for airway obstruction, such as common dysmorphic features (e.g., macroglossia and adenotonsillar hypertrophy), obesity, hypotonia, and gastroesophageal reflux (50–52). Intervention options for OSA usually include adenotonsillectomy and then continuous positive airway pressure therapy for individuals displaying residual OSA (53). However, adenotonsillectomy appears to be less effective in treating OSA in children with DS (54, 55). The persistence of sleep apneas in children with DS who had undergone adenotonsillectomy could be also explained by physical features typical of the syndrome, such as macroglossia and glossoptosis (54). Thus, the slight decrease in SBD scores we observed in adolescents with DS is unsurprising. This is consistent with a previous research report: in a sample of 255 individuals with DS, 47.5% of children who had undergone adenotonsillectomy had witnessed apnea (56). Altogether, these findings highlight the importance of continuous monitoring of SBD throughout the entire development of young people with DS.

Finally, poor differences between age groups in the percentage of clinical/borderline scores at DIMS scale emerged. These findings are not consistent with literature on general population, for which an increased rate of insomnia in adolescence has been reported. For instance, a large population-based study involving 611 participants at two time points (9 and 13 years of age, respectively) observed an increased prevalence of chronic insomnia from 4.2% (baseline) to 6.6% (follow-up) (57). More generally, adolescents tend to exhibit higher levels of sleep deprivation (58–60). The lack of important differences in the prevalence of difficulties in initiating and maintaining sleep between children and adolescents may indicate that these kinds of sleep problems tend to remain stable in the transition to adolescence, likely as disorders associated with the possible presence of OSA or with behavioral problems. Indeed, DIMS in children and adolescents with DS have been previously found to be associated with a range of emotional and behavioral problems, such as inattention and hyperactivity (33).

Previous studies concerning sex differences in sleep difficulties in children and adolescents has provided mixed findings. The lack of sex difference on the SDSC scores we observed in the school-age group is consistent with previous research focusing exclusively on preadolescents that reported no or scarce sex differences in sleep difficulties in school-age children with DS (42, 61). Conversely, other studies that have included participants with a wider age range have reported higher prevalence of SBD-related problems in males (62). Therefore, inconsistent results in literature could be due, at least in part, to methodological differences linked with the age range of the participants included. As our results suggest, one could speculate that sex differences in SBD-related problems are more likely to manifest in adolescence. Further research is required to investigate factors contributing to possible sex differences in SBD-related disorders in youth with DS.

The second aim of the study was to investigate the existence of specific age-based associations between sleep problems and emotional/behavior problems in children vs. adolescents with DS. In both groups, scores at the DIMS scale were significantly associated with emotional and behavioral problems as detected through the CBCL. Few group differences emerged in the pattern of associations between DIMS and behavioral problems; the most relevant one concerns the relationship between withdrawn and depressive symptoms. Indeed, in school-age children, such association did not emerge at all, whereas adolescents exhibited a strong relationship between DIMS and the Withdrawn/Depressed scale of the CBCL. This is consistent with research on TD, suggesting that difficulties initiating and maintaining sleep could be a “red flag” for depressive symptoms (63). This finding can have intriguing implications for the clinical practice, suggesting that the presence of difficulties in initiating and/or maintaining sleep could be considered a useful clinical indicator of the presence of internalizing problems in adolescents with DS. This is crucial, as adolescents with DS are at risk for internalizing issues (35). Clinicians should provide proper psychoeducation to caregivers, underlining the importance of monitoring the presence of sleep difficulties in adolescence.

Significant group differences also emerged in the patterns of association between sleep-related breathing difficulties and emotional and behavioral problems. Children exhibited associations between SBD scores and Somatic Complaints and Attention Problems scales of the CBCL, whereas in adolescents a relationship between SBD and any CBCL scale did not emerge. The association between sleep difficulties related to respiratory problems and the Somatic Complaints scale of the CBCL has been previously reported in a sample of children and adolescents without developmental delays aged 3–18 years (64). However, the association between sleep-related breathing problems and neurocognitive problems, including attention difficulties, has been previously reported in both typically developing population (65, 66) and DS (32); intriguingly, Brooks et al. (67) reported amelioration of parent-reported attention problems after treatment of SBD. However, inconsistent results emerged for DS population (33, 61, 66). The contrasting findings about the association between attention problems and sleep-related breathing problems could be due to, at least in part, the methodological differences among studies, related to the age of participants included and the assessment of SBD and/or behavioral problems. To the best of our knowledge, the current study is the first investigating this association in a large sample of individuals with DS by distinguishing between school-age children and adolescents. Indeed, the relationships between sleep-related breathing problems and behavioral difficulties have been poorly investigated by studies specifically focusing on adolescents with DS. The lack of association between SBD and behavior is consistent with earlier research including a sample with a wide age range (33). It can be speculated that behavioral changes occurring in adolescents with DS (35–37) may introduce some confounding factors masking the effects of sleep-related breathing problems in this population. Further research is needed to identify possible mediating factors.

In line with expectations based on evidence that parasomnias are relatively frequent conditions in childhood, poor associations between arousal disorders and emotional and behavior problems emerged at school age. We only observed a positive correlation with the Somatic Complaints scale of the CBCL. This is consistent with previous findings linking parasomnias and, in particular, sleep bruxism with somatic complaints but not with anxiety in school-age children (68). Similarly, a subsequent study detected an association between night terrors and the Somatic Complaints scale of the CBCL (69). As the association with the Somatic Complaints scale has been observed for almost all the SDSC scales, our results further support, in DS population, the link between sleep problems and somatic complaints observed in previous studies that found an increased number of sleep problems in children with somatic disorders (70, 71). However, in adolescents DA scores correlated positively with Thought Problems and Attention Problems. This suggests emerging or persisting arousal disorders in adolescents with DS should be carefully evaluated within an accurate global neuropsychiatric examination taking into account also emotional and behavioral domains.

Sleep problems associated with both sleep–wake transitions and excessive daily somnolence seem to be generally related to emotional and behavioral problems (both internalizing and externalizing) across school age and adolescence. This finding reaffirms the importance of taking into proper account the presence of sleep difficulties in children exhibiting behavioral problems—and vice versa—in psychological and neuropsychiatric assessment of youth with DS. Excessive daily somnolence could be regarded as a feature of behavioral problems, biological sleep disorders, other medical and psychiatric conditions, and as a risk factor for poor cardiovascular, neurological, and psychiatric outcomes (72). Given such a complex connection with different medical conditions, multidisciplinary approaches for clinical care are highly required, in particular for individuals with DS that are prone to several medical comorbidities. Of note, the clinical manifestations of excessive somnolence can differ between patients, presenting, for instance, with a continuous state of persistent somnolence or with a sudden overwhelming sleepiness occurring without antecedent signals (72). Therefore, clinicians should properly educate caregivers in detecting signals of ongoing sleep difficulties in their children with DS to timely identify potential problematic situations and ensure opportune intervention.

Our results generally corroborated findings on the presence of differential patterns of association between sleep difficulties and emotional and behavioral problems across different age groups. In particular, among school-age children, participants with sleep problems scored significantly higher in the CBCL scales detecting difficulties associated with Somatic Complaints, Thought and Attention Problems, and Rule-Breaking Behavior. However, the presence of a group effect among adolescents indicates that sleep difficulties could be considered general signals of emotional and behavioral problems in adolescents with DS.

There are a number of mechanisms proposed to explain the association between sleep and behavior problems; for instance, impaired synaptic plasticity could have negative consequences on memory and other neurocognitive functions (73, 74), whereas altered connectivity between the amygdala and the medial–prefrontal cortex could impact emotion regulation processes (75). Adding to the complexity of the picture is the role of factors that might contribute to the association between sleep difficulties and behavioral problems. Among these, a non-secondary role is the one that is played by sensory hypersensitivity, especially for children and adolescents with neurodevelopmental disorders. It has been proposed, indeed, that sensory processing abilities contribute to the relationship between sleep and behavior (76). Moreover, research in autism spectrum disorder showed that sleep problems are associated with heightened sensory sensitivity in this population (77–79). In particular, Mazurek et al. reported sensory over-responsivity as a longitudinal predictor of sleep difficulties and inattention/hyperactivity in very young but not in older children with autism spectrum disorder (80). Future research on the DS population should include the evaluation of sensory over-responsivity to explore its potential role as a contributor to the association between sleep difficulties and behavior across different developmental stages.

Limitations of the study include the cross-sectional nature of the study. A better understanding of the developmental trajectories of sleep problems requires longitudinal studies on large samples to explore the temporal link between sleep problems and behavior in youth with DS. Despite literature supporting the use of SDSC for children with DS (81), another important limitation of the study is the lack of objective measures to investigate sleep disorders in our sample. Future research should explore the association between sleep disorders—as measured by objective instruments—and behavioral problems in large samples of children and adolescents with DS. Similarly, the mono-method approach employed in the current study for behavioral evaluation only used parent reports of participants' behavior difficulties. The employment of other kinds of instruments, such as semi-structured diagnostic interviews, could provide a more reliable assessment of behavioral and emotional problems.

Despite these limitations, the present study provides new insights into the prevalence and distribution of sleep difficulties and their association with emotional and behavioral problems in pediatric populations with DS.

## Conclusions

Transition to adolescence is a crucial changeover for individuals with DS. The presence of differential patterns of association between sleep difficulties and emotional and behavioral problems across different age groups remarks the importance of appropriate neuropsychiatric and psychological evaluation by taking into account the age-specific needs and features of individuals with DS.

## Data availability statement

The raw data supporting the conclusions of this article will be made available by the authors, without undue reservation.

## Ethics statement

Ethical review and approval was not required for the study on human participants in accordance with the local legislation and institutional requirements. Written informed consent to participate in this study was provided by the participants' legal guardian/next of kin.

## Author contributions

EF, FC, DV, and SV: conceptualization and writing—review and editing. EF and FC: methodology, formal analysis, and writing—original draft preparation. EF, LC, and PG: investigation. LC and PG: data curation. AV and SV: supervision. SV: project administration. All authors have read and agreed to the published version of the manuscript. All authors contributed to the article and approved the submitted version.
